# Mass Spectrometric Proteomics 2.0

**DOI:** 10.3390/ijms25052960

**Published:** 2024-03-04

**Authors:** Paolo Iadarola, Simona Viglio

**Affiliations:** 1 Department of Biology and Biotechnologies “L.Spallanzani”, University of Pavia, 27100 Pavia, Italy; 2 Department of Molecular Medicine, Biochemistry Unit, University of Pavia, 27100 Pavia, Italy; simona.viglio@unipv.it; 3 Lung Transplantation Unit, IRCCS Policlinico San Matteo Foundation, 27100 Pavia, Italy

This Special Issue, “Mass Spectrometric Proteomics 2.0”, presents the second volume of a series dedicated to the dissemination of results obtained from the application of MS-based proteomics across different areas. Although not all problems in MS proteomics have been solved, this is currently a mature technique that has prompted the publication of thousands of articles in the biochemical sector to date [1]. That being said, the question arises as to whether the release of a “new” series on proteomics would make sense. A careful reading of this Special Issue suggests that it can rightly find its own place in the international literature among the journals dedicated to proteomics. The contributions that this volume has attracted have dealt with such diverse fields of life science research that they confirm, if required, the leadership of MS-based proteomics in handling the complexity of biological challenges that other methods cannot manage. Flexibility, reliability, and speed of execution are the three fundamental features of proteomics that have emerged from these papers. Interestingly, most of these reports are not purely focused on cataloging the total proteins present in any given samples but also provide new insights into the role played by some proteins in specific human disorders. This provides proof that proteomics has outgrown its infancy and entered the era of “systems biology”. Given its unique characteristics, proteomics, in synergy with other complementary methods (i.e., transcriptomics and metabolomics), makes it possible to obtain a global and integrated view of biological questions [2]. Another aspect that has not been neglected in this volume is the pivotal role played by proteomics in the area of biomarker discovery for the early diagnosis of human disorders. On the assumption that modifications occurring in an organism in response to different stimuli can be mirrored (to some degree) by changes in protein profiles, investigating how proteins are modulated between different conditions may shed light on the biological mechanisms involved in these processes [3,4]. The area focused on the investigation by MS of the proteome dynamics over time has witnessed enormous progress in recent years [5,6]. A crucial role in MS proteomics is also played by the availability of sophisticated methodological strategies to explore the molecular mechanisms behind different human disorders on a deeper level. In this context, an article that delves into the development of a new software tool for the visualization and validation of protein turnover rates must be acknowledged [7]. 

Given these premises, we hope that this new volume, “Mass Spectrometric Proteomics 2.0”, will soon be published.
